# Atrial signal amplitude predicts atrial high‐rate episodes in implantable cardioverter defibrillator patients: Insights from a large database of remote monitoring transmissions

**DOI:** 10.1002/joa3.12319

**Published:** 2020-03-02

**Authors:** Massimo Zecchin, Francesco Solimene, Antonio D'Onofrio, Gabriele Zanotto, Saverio Iacopino, Carlo Pignalberi, Valeria Calvi, Giampiero Maglia, Paolo Della Bella, Fabio Quartieri, Antonio Curnis, Mauro Biffi, Alessandro Capucci, Fabrizio Caravati, Gaetano Senatore, Matteo Santamaria, Fabio Lissoni, Michele Manzo, Massimiliano Marini, Massimo Giammaria, Antonio Rapacciuolo, Gianfranco Sinagra, Daniele Giacopelli, Alessio Gargaro, Ennio C. Pisanò

**Affiliations:** ^1^ Azienda Sanitaria Universitaria Integrata Trieste Italy; ^2^ Clinica Montevergine Mercogliano Italy; ^3^ Ospedale Monaldi Naples Italy; ^4^ Ospedale Mater Salutis Legnago Italy; ^5^ Villa Maria Care & Research Cotignola Italy; ^6^ Ospedale San Filippo Neri Rome Italy; ^7^ Policlinico G. Rodolico, Az. O.U. Policlinico ‐ V. Emanuele Catania Italy; ^8^ Azienda Ospedaliera Pugliese Ciaccio Catanzaro Italy; ^9^ Ospedale San Raffaele Milan Italy; ^10^ Arcispedale Santa Maria Nuova Reggio Emilia Italy; ^11^ Spedali Civili Brescia Italy; ^12^ Policlinico Sant'Orsola‐Malpighi Bologna Italy; ^13^ Ospedali Riuniti Ancona Italy; ^14^ Ospedale di Circolo e Fond. Macchi Varese Italy; ^15^ Ospedale di Ciriè Ciriè Italy; ^16^ Fondazione di Ricerca e Cura Giovanni Paolo II Campobasso Italy; ^17^ Ospedale di Lodi Lodi Italy; ^18^ Azienda Ospedaliera Universitaria S.Giovanni di Dio e Ruggi D'Aragona Salerno Italy; ^19^ Ospedale Santa Chiara Trento Italy; ^20^ Ospedale Maria Vittoria Torino Italy; ^21^ Azienda Ospedaliera Universitaria Federico II Naples Italy; ^22^ BIOTRONIK Italia Vimodrone Italy; ^23^ Ospedale Vito Fazzi Lecce Italy

**Keywords:** cardiac resynchronization therapy, impedance, implantable cardioverter defibrillator, pacing threshold, sensing

## Abstract

**Background:**

Parameters measured during implantable cardioverter defibrillator (ICD) implant also depend on bioelectrical properties of the myocardium. We aimed to explore their potential association with clinical outcomes in patients with single/dual‐chamber ICD and cardiac resynchronization therapy defibrillator (CRT‐D).

**Methods:**

In the framework of the Home Monitoring Expert Alliance, baseline electrical parameters for all implanted leads were compared by the occurrence of all‐cause mortality, adjudicated ventricular arrhythmia (VA), and atrial high‐rate episode lasting ≥24 hours (24 h AHRE).

**Results:**

In a cohort of 2976 patients (58.1% ICD) with a median follow‐up of 25 months, event rates were 3.1/100 patient‐years for all‐cause mortality, 18.1/100 patient‐years for VA, and 9.3/100 patient‐years for 24 h AHRE. At univariate analysis, baseline shock impedance was consistently lower in groups with events than without, with a 40 Ω cutoff that better identified high‐risk patients. However, at multivariable analysis, the adjusted‐hazard ratios (HRs) lost statistical significance for any endpoint. Baseline atrial sensing amplitude during sinus rhythm was lower in patients with 24 h AHRE than in those without (2.45 [IQR: 1.65‐3.85] vs 3.51 [IQR: 2.37‐4.67] mV, *P* < .01). The adjusted HR for 24 h AHRE in patients with atrial sensing >1.5 mV vs those with values ≤1.5 mV was 0.52 (95% CI: 0.33‐0.83), *P* = .006.

**Conclusions:**

Although lower baseline shock impedance was observed in patients with events, the association lost statistical significance at multivariable analysis. Conversely, low sinus rhythm atrial sensing (≤1.5 mV) measured with standard transvenous leads could identify subjects at high risk of atrial arrhythmia.

## Graphical abstract

Some of the electrical parameters routinely measured at ICD/CRT‐D implant differed according to long‐term clinical outcome. Shock impedance values were lower in patients who experienced death and both atrial and ventricular arrhythmias during follow‐up. Subjects with atrial signal amplitude below 1.5 mV showed a significant higher risk of atrial arrhythmias as compared to those with >1.5 mV, potentially revealing the presence of a more impaired atrial tissue.

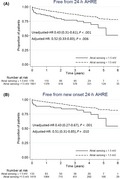

## 1 INTRODUCTION

During implant of implantable cardioverter defibrillators (ICDs) and cardiac resynchronization therapy defibrillators (CRT‐Ds), pacing threshold, impedance, and sensing amplitude are routinely assessed for all implanted leads. Their monitoring during follow‐up with regular in‐office visits or remote control is then used for the surveillance of integrity and functioning of leads.

Beyond technical aspects, several factors may influence these measurements, including properties of the myocardial tissue surrounding the lead electrodes.^1^, ^2^ Prior studies suggested an association between temporal changes in some of these parameters and clinical events, such as heart failure functional class changes or cardiac arrhythmias occurrence.^3^, ^4^, ^5^ However, it is unknown whether their values at implant could have a clinical relevance as a systematic analysis of association between baseline measurements and clinical outcomes has never been performed.

The aim of the present study was to explore whether baseline electrical parameters routinely measured during implant have an association with long‐term mortality or incidence of atrial and ventricular arrhythmias (VAs) in ICD/CRT‐D recipients.

## 2 METHODS

The present analysis was performed in the framework of the Home Monitoring Expert Alliance (HMEA), an independent scientific project based on a nationwide repository of data generated by remote monitoring (RM) of cardiac implantable electronic devices (CIEDs) during ordinary medical practice.^6^ A total of 41 Italian sites, listed in the Appendix, provided data for this analysis. All included patients provided written informed consent before RM activation.

### 2.1 Objective and patient selection

We aimed to investigate whether baseline electrical parameters routinely measured during CIED implant could show an association with long‐term mortality or incidence of atrial and VAs.

All ICD and CRT‐D recipients registered in the HMEA database were selected for the present analysis. Devices were implanted from January 2007 to March 2017.

### 2.2 Data collection and analysis endpoints

Baseline data were collected at the time of device implant. They included patient characteristics and electrical parameters routinely measured for any implanted lead during ICD/CRT‐D implant procedures. Pacing impedance, capture threshold, and sensing amplitude were collected for atrial, right ventricle (RV), and left ventricle (LV) leads. Electrical parameters were usually obtained in bipolar configuration, expect for a minority (2.7%) of unipolar LV leads. In addition, high‐voltage shock impedance, measured between distal coil and case, was reported for all RV leads. Atrial parameters were not included in the present analysis if the patient was in atrial fibrillation (AF) at implant.

All CIEDs were manufactured by the same company (Biotronik) and follow‐up data were automatically and daily generated by the RM system (Home Monitoring; Biotronik), which provided device diagnostics and intracardiac electrogram (IEGM) recordings of all atrial and ventricular arrhythmic episodes. Atrial high‐rate episodes (AHREs) were recorded and transmitted based on a rate criterion set at 200 bpm (standard setting of devices). The atrial sensitivity adapted automatically on an ongoing basis to the measured amplitude of the atrial activity. The lowest sensing threshold that can be reached was 0.1 mV. VAs were automatically classified by the discrimination algorithms of the device. The median detection cutoff programed for the first VA zone was 158 (IQR: 150‐171) beats/min with a counter of 28 (IQR: 26‐40) beats to detect. In order to exclude false episodes from the analysis, the first VA detected by the device was adjudicated by a three‐member board who visually reviewed IEGM recordings. A two‐stage adjudication process was used: a first stage used an algorithm of objective electrophysiological criteria such as ventricular cycle <atrial cycle; atrioventricular decoupling; unstable ventricular rhythm during atrial tachyarrhythmia. The second stage consisted of a majority vote, when the objective criteria were uncertain. In the event of an inappropriate detection, the search continued until detection of the first true VA or the last available RM transmission.

The endpoints of the analysis were time to all‐cause death and to first postimplant adjudicated VA and AHRE lasting ≥24 hours (24 h AHRE).

All‐cause mortality was estimated after site staff confirmed death status of patients with interrupted RM transmissions and no evidence of device replacement.

For the AHRE analysis, the 24 hour duration threshold was used because it could be the sign of impaired atrial tissue and has recently been reported as the duration associated with an increased risk of ischemic stroke or systemic embolism.^7^ Short‐lasting atrial arrhythmias seem to have less clinical significance and an immediate anticoagulation in these patients is unlikely to result in reduction of the risk of stroke.^8^ Only patients who had atrial diagnostics capability were selected for this endpoint.

In order to investigate whether some of the baseline electrical parameters could be a marker of endpoint occurrence, variables that had significant differences in the descriptive analysis were used to stratify event rates by value classes. The endpoints were then compared between the two subgroups defined using the value that maximized the difference in event rate as the cutoff value.

### 2.3 Statistical analysis

We described the selected population by using all‐cause death, VA, and 24 h AHRE occurrence as grouping criteria. Binary and categorical variables were reported as percentages of available data, and continuous variables as median (interquartile range [IQR]). Baseline between‐group comparisons were performed with the Wilcoxon signed‐rank test for continuous variable, Pearson chi‐squared or Fisher's tests for noncontinuous variables, as appropriate. Event rates were reported as the number of events divided by the amount of person‐time observed; the 95% confidence intervals (CIs) were calculated by means of the Poisson distribution within an iterative procedure. Kaplan‐Meier curves were generated and compared between groups using adjusted and unadjusted proportional hazard models. Adjusting covariates were age, sex, presence of hypertension, diabetes, ischemic cardiomyopathy, history of AF, and CHA_2_DS_2_‐VASC score. Statistical significance was defined as *P* < .05. All statistical analyses were performed using the version 11E of STATA software (StatCorp LB).

## 3 RESULTS

### 3.1 Population

A total of 2976 patients were included in the present analysis, 827 (27.8%) implanted with a single‐chamber ICD, 902 (30.3%) with a dual‐chamber ICD, and 1247 (41.9%) with a CRT‐D device. Baseline characteristics are reported in Table 1.

**TABLE 1:** Patient characteristics

	Total	Survivors	Deceased	*P* *	Free from VA	VA	*P* *	Free from 24 h AHRE	24 h AHRE	*P* *
Number of patients	2976	2849	227	–	2022	954	–	1998	500	–
Follow‐up (mo)	25 [12‐44]	25 [12‐44]	25 [12‐44]	.55	21 [10‐37]	37 [22‐51]	<.01	24 [12‐42]	26 [14‐47]	.01
Age (y)	70 [61‐77]	69 [60‐77]	75 [69‐80]	<.01	70 [31‐77]	69 [61‐77]	.43	69 [60‐76]	74 [67‐79]	<.01
Sex (female)	19.4%	19.4%	19.8%	.86	20.9%	16.4%	.01	20.9%	16.0%	.02
NYHA functional class										
I‐II	71.3%	73.5%	48.2%	<.01	72.1%	69.6%	.56	72.7%	66.1%	<.01
III‐IV	27.5%	25.5%	49.4%		26.8%	29.1%		27.3%	33.9%	
LVEF, %	30 [27‐35]	30 [28‐35]	30 [25‐35]	.01	30 [28‐35]	30 [25‐35]	.97	30 [27‐35]	30 [28‐35]	.95
QRS duration (ms)	120 [100‐140]	120 [100‐140]	130 [120‐150]	<.01	120 [100‐140]	120 [100‐140]	.89	120 [100‐143]	130 [106‐145]	.05
Device type										
Single chamber ICD	27.8%	28.2%	23.3%	.01	27.9%	27.0%	.83	–	–	<.01
Dual‐chamber ICD	30.3%	30.9%	22.9%		30.2%	33.0%		57.5%	46.7%	
CRT‐D	41.9%	40.9%	53.7%		42.0%	40.0%		42.5%	53.3%	
Comorbidities										
Hypertension	52.4%	52.7%	48.4%	.26	53.1%	51.0%	.35	52.6%	49.5%	.26
Diabetes	23.6%	23.1%	29.8%	.04	25.2%	20.2%	.01	23.7%	24.1%	.88
Stroke/TIA	8.7%	7.9%	11.3%	.10	8.0%	8.7%	.53	8.8%	7.9%	.56
Chronic kidney disease	13.0%	11.7%	27.5%	<.01	13.1%	12.6%	.73	12.6%	15.1%	.19
History of heart failure	23.4%	22.9%	32.5%	.02	24.6%	20.5%	.04	22.2%	22.8%	.82
CHA_2_DS_2_‐VASC class										
0‐1	10.2%	10.9%	0.6%	<.01	9.4%	12.0%	.39	10.9%	6.4%	<.01
2	16.2%	17.0%	7.0%		17.3%	14.0%		17.1%	13.1%	
3	23.4%	23.3%	24.0%		23.3%	23.4%		23.3%	21.3%	
4	24.9%	24.4%	30.4%		24.1%	26.4%		24.3%	23.2%	
≥5	25.3%	24.4%	38.0%		25.9%	24.2%		24.4%	36.0%	
Cardiomyopathy										
Ischemic	50.8%	49.8%	61.8%	.01	51.0%	50.3%	.74	51.4%	53.8%	.41
Dilated idiopathic	35.2%	35.6%	30.4%	.15	34.6%	36.3%	.43	35.6%	33.9%	.52
Valvular	7.7%	7.6%	8.5%	.66	6.8%	9.7%	.01	5.9%	8.6%	.07
Other	5.8%	6.0%	2.1%	.24	6.5%	4.2%	.04	4.9%	5.9%	.85
Documented arrhythmias										
Ventricular fibrillation	7.8%	8.2%	3.2	.02	6.8%	9.9%	.01	7.6%	6.3%	.37
Sustained VT	16.1%	15.8%	19.9%	.14	12.3%	24.5%	<.01	15.0%	13.8%	.55
History of AF	21.6%	20.9%	29.1%	.01	20.1%	24.7%	.01	9.5%	49.8%	<.01
Paroxysmal AF	7.0%	6.7%	9.9%		7.1%	6.7%		7.0%	13.2%	
Persistent/permanent AF	14.6%	14.2%	19.2%		13.0%	18.0%		2.5%	36.6%	

Note

Data are reported as median [interquartile range] or percentage.

Only devices with atrial sensing capability were included in the 24 h AHRE analysis.

Abbreviations: AF, atrial fibrillation; AHRE, atrial high‐rate episode; CRT‐D, cardiac resynchronization therapy‐defibrillator; ICD, implantable cardioverter defibrillator; LVEF, left ventricular ejection fraction; TIA, transient ischemic attack; VA, ventricular arrhythmias; VT, ventricular tachycardia.

* Wilcoxon signed‐rank test or Pearson χ^2^.

During a median follow‐up of 25 [IQR: 12‐44] months, there were 227 (7.6%) all‐cause deaths (event rate: 3.1/100 patient‐years). As expected, survivors were younger, had lower New York Heart Association (NYHA) functional class and QRS duration, and higher left ventricular ejection fraction.

Adjudicated VAs were found in 954 (32.0%) patients (event rate: 18.1/100 patient‐years), more frequently in males and in subjects implanted for secondary prevention and with history of AF.

Among the 2498 patients implanted with devices with atrial sensing capability, 500 (20.0%) developed 24 h AHRE (event rate: 9.3/100 patient‐years). The group with atrial arrhythmia had higher age, lower female prevalence and, as expected, a very significant proportion (49.8%) of patients with history of AF before enrollment (despite in sinus rhythm at implant).

### 3.2 Baseline electrical parameters and all‐cause death

Some of the baseline electrical parameters were statistically different between survivors and deceased patients (Table 2). Atrial sensing (3.38 [IQR: 2.30‐4.61] mV vs 2.63 [IQR: 1.74‐3.86] mV, *P* < .01), LV pacing impedance (621 [IQR: 498‐749] Ω vs 579 [IQR: 460‐695] Ω, *P* = .01), and LV sensing (12.0 [IQR: 8.32‐16.5] Ω vs 10.7 [IQR: 8.09‐12.8] Ω, *P* = .02) were slightly higher in survivors. A more marked difference was observed in the shock impedance with a median value of 61 [IQR: 52‐69] Ω in the survivors compared to 51 [IQR: 41‐61] Ω in deceased subjects (*P* < .01).

**TABLE 2:** Baseline electrical parameters by groups

	Total	Survivors	Deceased	*P* *	Free from VA	VA	*P* *	Free from 24 h AHRE	24 h AHRE	*P* *
Atrial pacing impedance (Ω)^†^	554 [487‐645]	554 [489‐644]	542 [463‐673]	.53	554 [491‐637]	552 [476‐668]	.84	554 [488‐646]	549 [480‐641]	.39
Atrial threshold (V)^†^	0.80 [0.60‐1.02]	0.80 [0.60‐1.02]	0.80 [0.60‐1.02]	.93	0.85 [0.64‐1.06]	0.80 [0.60‐1.00]	**.03**	0.80 [0.60‐1.03]	0.81 [0.65‐1.02]	.16
Atrial sensing (mV)^†^	3.33 [2.26‐4.58]	3.38 [2.30‐4.61]	2.63 [1.74‐3.86]	**<.01**	3.44 [2.30‐4.66]	3.18 [2.19‐4.34]	**.01**	3.51 [2.37‐4.67]	2.45 [1.65‐3.85]	**<.01**
RV pacing impedance (Ω)	524 [469‐595]	524 [470‐595]	515 [452‐605]	.38	524 [470‐594]	524 [468‐603]	.69	522 [470‐592]	516 [464‐592]	.35
RV threshold (V)	0.60 [0.50‐0.77]	0.60 [0.50‐0.77]	0.61 [0.50‐0.92]	.14	0.60 [0.50‐0.77]	0.60 [0.49‐0.77]	.94	0.61 [0.50‐0.78]	0.60 [0.50‐0.80]	.86
RV sensing (mV)	11.7 [8.48‐15.9]	11.7 [8.48‐16.1]	11.4 [9.16‐14.7]	.41	12.1 [8.63‐16.5]	11.1 [8.24‐14.3]	**<.01**	11.9 [8.65‐16.3]	11.0 [8.19‐14.6]	**<.01**
LV pacing impedance (Ω)	616 [494‐743]	621 [498‐749]	579 [460‐695]	**.01**	606 [486‐741]	633 [522‐750]	**.03**	610 [492‐738]	648 [525‐793]	**.01**
LV threshold (V)	1.01 [0.70‐1.42]	1.02 [0.71‐1.42]	0.98 [0.62‐1.48]	.54	1.01 [0.73‐1.44]	1.01 [0.66‐1.35]	.26	1.10 [0.75‐1.59]	1.01 [0.80‐1.50]	.70
LV sensing (mV)	11.8 [8.25‐15.9]	12.0 [8.32‐16.5]	10.7 [8.09‐12.8]	**.02**	12.2 [8.25‐16.9]	11.1 [8.33‐14.4]	**.01**	12.0 [8.29‐16.8]	11.6 [8.90‐14.6]	.17
Shock impedance (Ω)	61 [51‐69]	61 [52‐69]	51 [41‐61]	**<.01**	62 [53‐70]	56 [46‐65]	**<.01**	62 [52‐70]	56 [46‐66]	**<.01**

Note

Data are reported as median [interquartile range].

Only devices with atrial sensing capability were included in the 24 h AHRE analysis.

Abbreviations as listed in Table 1.

* Wilcoxon signed‐rank test. Bold values if *p* < .05.

^†^ Excluding patients in atrial fibrillation at implant.

### 3.3 Baseline electrical parameters and ventricular arrhythmias

Patients without adjudicated VA had slightly higher atrial threshold (0.85 [IQR: 0.64‐1.06] vs 0.80 [IQR: 0.60‐1.00] V, *P* = .03), atrial signal amplitude (3.44 [IQR: 2.30‐4.66] mV vs 3.18 [IQR: 2.19‐4.34] mV, *P* = .01), RV signal amplitude (12.1 [IQR: 8.63‐16.5] mV vs 11.1 [IQR: 8.24‐14.3] mV, *P* < .01), and LV signal amplitude (12.2 [IQR: 8.25‐16.9] mV vs 11.1 [IQR: 8.33‐14.4] mV, *P* = .01) as compared to patients who experienced VAs. Shock impedance was still different between groups confirming a lower value in patient who experienced this endpoint (56 [IQR: 46‐65] Ω vs 62 [IQR: 53‐70] Ω, *P* < .01). On the other hand, baseline LV pacing impedance was lower in subjects free from VA (606 [486‐741] Ω vs 633 [522‐750] Ω, *P* = .03). Table 2 depicts the entire analysis.

### 3.4 Baseline electrical parameters and atrial arrhythmias

When considering 24 h AHRE, few baseline electrical parameters showed differences between groups. Lower values of atrial signal amplitude (2.45 [IQR: 1.65‐3.85] mV vs 3.51 [IQR: 2.37‐4.67] mV, *P* < .01) and shock impedance (56 [IQR: 46‐66] Ω vs 62 [IQR: 52‐70] Ω, *P* < .01) were observed in patients who experienced 24 h AHRE. RV signal amplitude and LV pacing impedance values had minor differences (Table 2).

### 3.5 Markers of arrhythmia occurrence and prognosis

**FIGURE 1:**
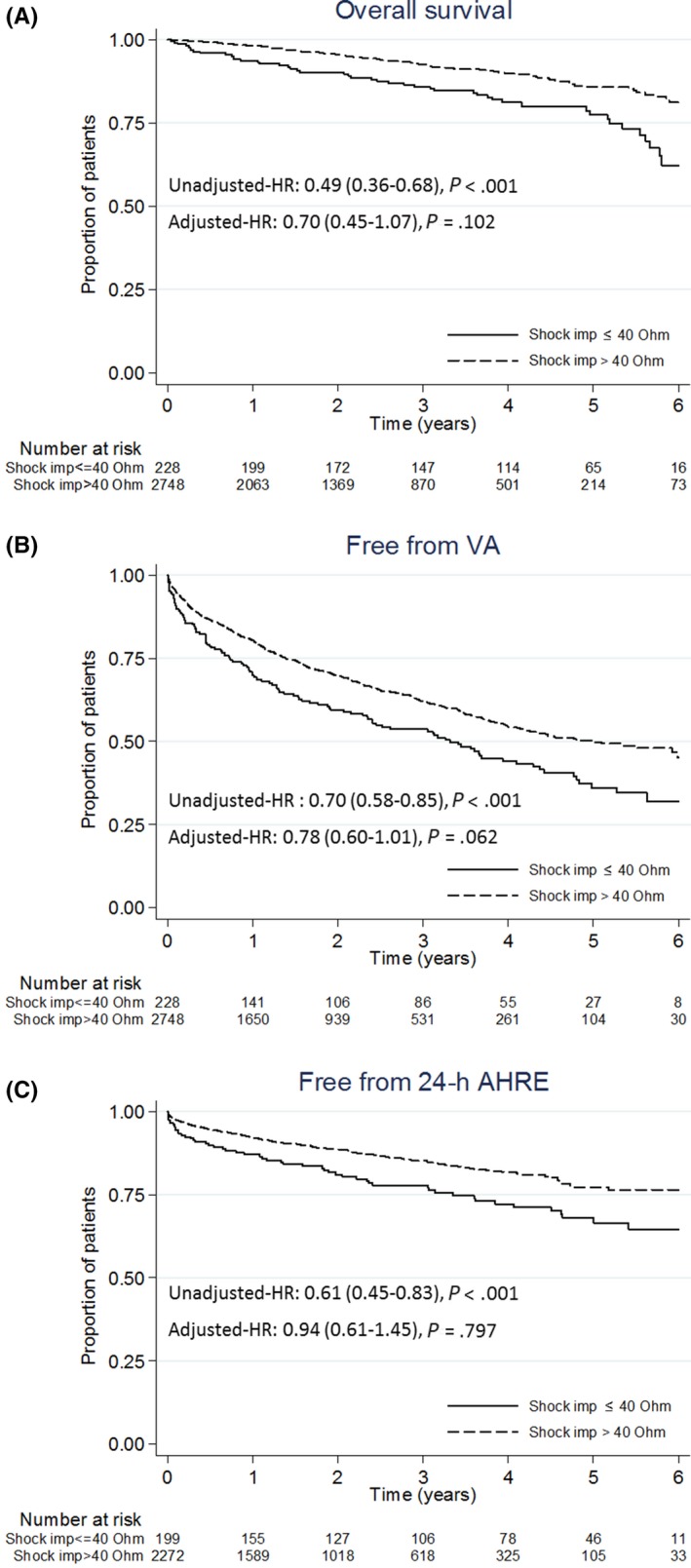
Kaplan‐Meier curves of all‐cause mortality (A), VA (B), and 24 h AHRE (C) occurrence free rates by ≤40 and >40 Ω baseline shock impedance. AHRE, atrial high‐rate episode; VA, ventricular arrhythmia

At the descriptive analysis, the baseline shock impedance showed consistent differences for all study endpoints with lower median values in patients who experienced death, VA, and AHRE. Table 3 reported the event rates in subgroups according to their baseline shock impedance value. Patients with shock impedance ≤40 Ω had a higher incidence of all study endpoints: 6.0 (95% CI: 4.5‐7.9)/100 patient‐years for all‐cause death, 22.8 (95% CI: 18.5‐27.2)/100 patient‐years for VA, and 10.8 (95% CI: 8.4‐13.8)/100 patient‐years for 24 h AHRE occurrence. Figure 1 shows Kaplan‐Meier curves: at 6 years, all‐cause mortality for this subgroup was 36.2% (95% CI: 27.0%‐47.4%) with 67.8% (95% CI: 59.1%‐76.2%) VA and 44.6% (95% CI: 35.6%‐54.8%) 24 h AHRE incidence. However, the adjusted HRs between patients with shock impedance >40 Ω vs those with values ≤40 Ω were not statistically significant (all‐cause mortality: 0.70 [0.45‐1.07], *P* = .102; VA: 0.78 [0.60‐1.01], *P* = .062; 24 h AHRE: 0.94 [0.61‐1.45], *P* = .797). Table 4 reports the association of the adjusting covariates of the multivariate models.

**TABLE 3:** Event rates of death, VA, and 24 h AHRE occurrence by baseline shock impedance subgroups

Shock impedance subgroup	Deaths	Event rate	VA	Event rate	24 h AHRE	Event rate
≤40 Ω	50	6.0 (4.5‐7.9)	119	22.8 (18.5‐27.2)	67	10.8 (8.4‐13.8)
>40 and ≤50 Ω	61	3.5 (2.7‐4.5)	213	18.3 (15.9‐20.9)	100	8.6 (6.9‐10.4)
>50 and ≤60 Ω	53	2.8 (2.1‐3.6)	259	18.4 (16.2‐20.8)	137	9.9 (8.3‐11.7)
>60 and ≤70 Ω	45	2.4 (1.8‐3.3)	248	17.8 (15.7‐20.2)	124	8.8 (7.3‐10.5)
>70 Ω	18	1.9 (1.1‐2.9)	115	14.6 (12.0‐17.5)	72	9.2 (7.2‐11.6)
Total	227	3.1 (2.7‐3.6)	954	18.1 (16.9‐19.3)	500	9.3 (8.5‐10.2)

Note

Event rates are expressed as events/100 patient‐years (95% confidence interval).

Only devices with atrial sensing capability were included in the 24 h AHRE analysis.

Abbreviations as listed in Table 1.

**TABLE 4:** Association between baseline shock impedance >40 Ω and all‐cause mortality, VA and 24 h AHRE incidence

	All‐cause mortality			VA			24 h AHRE		
	HR	95% CI	*P*	HR	95% CI	*P*	HR	95% CI	*P*
Unadjusted model									
Shock impedance >40 Ω	0.49	0.36‐0.68	<.001	0.70	0.58‐0.85	<.001	0.61	0.45‐0.83	.001
Adjusted model									
Shock impedance >40 Ω	0.70	0.45‐1.07	.102	0.78	0.60‐1.01	.062	0.94	0.61‐1.45	.797
Adjusting covariates									
Age	1.03	1.01‐1.05	.004	1.00	0.99‐1.01	.384	1.01	0.99‐1.03	.184
Sex (female)	1.16	0.76‐1.76	.489	0.77	0.62‐0.95	.016	0.56	0.37‐0.84	.005
Hypertension	0.76	0.54‐1.07	.113	0.96	0.81‐1.14	.630	0.75	0.56‐1.00	.050
Diabetes	1.58	1.10‐2.25	.012	0.84	0.69‐1.02	.129	0.96	0.70‐1.32	.803
Ischemic cardiomyopathy	1.39	0.93‐2.07	.111	0.91	0.76‐1.10	.347	0.97	0.71‐1.34	.869
History of AF	1.47	1.03‐2.12	.034	1.30	1.08‐1.57	.006	3.72	2.80‐4.94	<.001
CHA_2_DS_2_‐VASC score	1.15	0.98‐1.36	.093	1.05	0.97‐1.14	.255	1.15	1.00‐1.31	.042

Note

Univariate and multivariate Cox proportional hazard models.

Only devices with atrial sensing capability were included in the 24 h AHRE analysis.

Abbreviations: AF, atrial fibrillation; AHRE, atrial high‐rate episode; CI, confidence interval; HR, hazard ratio; VA, ventricular arrhythmias.

**FIGURE 2:**
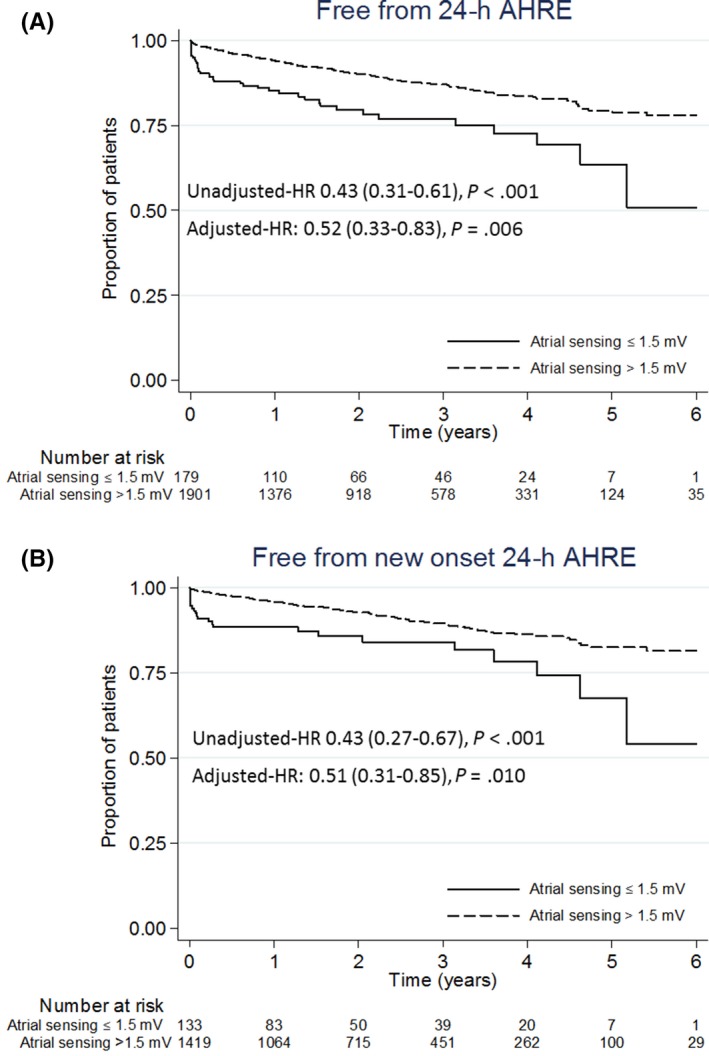
Kaplan‐Meier curves of AHRE lasting >24 h occurrence free rates by ≤1.5 and >1.5 mV baseline atrial sensing for all patients (A) and after excluding patients with history of atrial fibrillation (B). Patients in atrial fibrillation at implant were excluded. AHRE, atrial high‐rate episode

The incidence of 24 h AHRE was significantly different according to baseline atrial signal amplitude measured in sinus rhythm (Table 5). Subjects with atrial signal ≤1.5 mV showed an event rate of 24 h AHRE of 23.3 (95% CI: 18.4‐29.2)/100 patient‐years, with an incidence of 37.1% (30.5%‐44.6%) and 60.8% (40.7%‐81.3%) at 2 and 6 years, respectively (Figure 2A). The adjusted HR of events in patients with atrial signal >1.5 mV vs those with values ≤1.5 mV was 0.52 (95% CI: 0.33‐0.83), *P* = .006 (unadjusted HR 0.43 [95% CI: 0.31‐0.61], *P* < .001).

**TABLE 5:** Event rates of 24 h AHRE occurrence by baseline atrial sensing (all patients in sinus rhythm at implant)

Atrial sensing subgroup	24 h AHRE	Event rate
≤1.5 mV	76	23.3 (18.4‐29.2)
>1.5 and ≤2.5 mV	102	9.6 (7.8‐11.6)
>2.5 and ≤3.5 mV	65	6.2 (4.8‐7.9)
>3.5 and ≤4.5 mV	46	4.4 (3.3‐5.9)
>4.5 mV	54	4.7 (3.5‐6.2)
Total	343	7.4 (6.7‐8.3)

Note

Event rates are expressed as events/100 patient‐years (95% confidence interval).

Only devices with atrial sensing capability. Patients in atrial fibrillation at implant were excluded.

Abbreviations as listed in Table 1.

After excluding patients with a known previous history of AF, atrial signal >1.5 mV confirmed to be significantly associated with a lower risk of 24 h AHRE during follow‐up as shown in Figure 2B (adjusted HR: 0.51 [95% CI: 0.31‐0.85], *P* = .010).

## 4 DISCUSSION

In the present analysis on about 3000 ICD and CRT‐D patients, we found some associations between electrical parameters at implant and long‐term clinical outcomes. Baseline shock impedance values were lower in patients with atrial and VAs and in those who died during follow‐up. A cutoff of 40 Ω identified a subgroup with a particularly high incidence of events; however, the association was not significant if adjusted by other patients' characteristics.

Conversely, the higher incidence of atrial arrhythmias in patients with baseline atrial sensing in sinus rhythm ≤1.5 mV compared to >1.5 mV was statistically significant even after adjustment with patient characteristics. This parameter could be used as a potential marker of underlying atrial tissue disease, potentially identifying patients who may benefit from an intensive monitoring approach which can be provided by daily RM.

### 4.1 Predictors of death and ventricular arrhythmias

Electrical data obtained during device implant can be influenced by several factors, as lead heart contact, lead position, and lead characteristics. However, bioelectrical properties can also be modified by other factors, including ischemia or the presence of fibrosis. In addition, shock impedance, which is calculated between the distal part of the lead (ventricular coil) and the ICD, depends on the conduction characteristics of the thorax including the whole heart and lungs. Therefore, low values can be observed when electrical conduction is favored, as in the case of fluid overload. As a result, deceased and VA/AHRE patients showed lower baseline shock impedance in our study, probably as a consequence of increased lung congestion and more severe heart failure symptoms even at implant. However, we were not able to detect a significant association in multivariable models between baseline values and events occurrence. The association between decreased shock impedance and heart failure or VA was shown in several studies, but the temporal relationship between the events is still unclear.^5^

We also found differences in the left ventricular pacing impedance, which is a near‐field measurement, as calculated mainly in bipolar configuration. Patients who died during follow‐up had lower baseline values than survivors. This result may reflect a higher percentage of patients with the LV lead located in an ischemic zone since infarct scar showed lower electrical impedance than the normal myocardium.^1^, ^9^ Finally, lower signal amplitudes for both right and LV leads were found in patients with death and VA events. This is not surprising as cytopenia and fibrosis are associated with lower signals and more advanced heart disease.^4^, ^10^ However, it should be noted that these parameters were statistically different only at the univariate analysis and the difference between groups was very small and with questionable clinical significance.

### 4.2 Predictors of AHRE

The difference of atrial signal amplitude at implant between patients with and without AHRE later detected during follow‐up was more striking.

It is well known that in the atria low signal amplitude is associated with the presence of scar,^9^ AF recurrences,^11^ and heart failure.^12^, ^13^ However, most data were obtained during atrial mapping for AF ablation, while data are lacking on how intraoperative atrial signal amplitude during sinus rhythm can predict atrial tachyarrhythmias after device implant.

In our study, patients with atrial signal amplitude lower than 1.5 mV at implant had a risk of 24 h atrial arrhythmias of 23.3/100 patient‐years during follow‐up, while for patients with higher atrial signal values the overall event rate was 6.2/100 patient‐years. This association was significant even if adjusted by other patients' characteristics and excluding patients with history of atrial arrhythmias before implant. AF is often asymptomatic and the identification of high‐risk patients is still an open issue. Recent data showed an incidence of 24 h AHRE in patients without AF history at high risk for thromboembolic events (CHA_2_DS_2_‐VASC score ≥ 5) of 7.7% and 40.4% at 2 and 6 years, respectively.^14^ In our analysis, at 2 and 6 years, 29.9% and 42.6% of patients with baseline low atrial signal developed this arrhythmia. In this scenario, atrial signal amplitude in sinus rhythm could be a useful marker to identify subjects more likely to develop atrial arrhythmias who may benefit from an intensive monitoring approach, which can be provided by daily RM.

More intriguing is the relationship found between AHRE and shock impedance, confirming the association between atrial arrhythmias and severity of heart disease,^15^ despite the difference, although statistically significant, was clinically quite negligible.

### 4.3 Limitations

This study is an observational retrospective analysis suffering from all the known limitations of this design. Leads were placed according to clinical practice without specific recommendations and were not verified with fluoroscopy images, excluding the use of a variable lead location as adjusting covariate in our models. However, the large sample size of the database in terms of patients and sites is an important strength of this analysis tempering potential biases.

As the HMEA database is based on the Home Monitoring system, all devices included in the present analysis were made by Biotronik and this could have an impact on the detection algorithms of AHRE, signals, and impedance measurements.

Atrial high‐rate episodes were not adjudicated potentially including far‐field artifact and noise. However, the impact of the adjudication has been shown to be less relevant when using relatively long thresholds for diagnosis. The positive predictive value of AHRE increased to 98.2% when the threshold duration was prolonged to 24 h as in our analysis.^16^

Finally, device programing was not uniform reflecting ordinary medical practice. As a relevant proportion of the devices included in our cohort were implanted before 2014 when more aggressive antitachycardia settings and shorter detections were largely used, the rate of VA may be higher as compared to contemporary cohorts.

## 5 CONCLUSIONS

Shock impedance values were lower in patients who experienced death and both atrial and VAs during follow‐up. However, the association was not significant if adjusted by other patients' characteristics. Conversely, subjects with atrial signal amplitude below 1.5 mV showed a significant higher risk of atrial arrhythmias as compared to those with >1.5 mV, potentially revealing the presence of a more impaired atrial tissue.

## CONFLICT OF INTERESTS

DG and AG are employees of BIOTRONIK Italia; the remaining authors have no conflict of interests for this article.

## Supporting information

Appendix
