# Supplementary material for: Atrial signal amplitude predicts atrial high‐rate episodes in implantable cardioverter defibrillator patients: Insights from a large database of remote monitoring transmissions
Source: J Arrhythm. 2020 Mar 2;36(2):353–62. doi: 10.1002/joa3.12319 (PMC7132187; doi:10.1002/joa3.12319)
Supplement: Supplementary file 1 — Appendix [file JOA3-36-353-s001.docx]

# Appendix

## List of HMEA centres and investigators.

AOU "G. Martino", Messina: Giuseppe Picciolo, Pasquale Crea; AOU Senese, Siena: Valerio Zacà, Claudia Baiocchi; Arcispedale Santa Maria Nuova, Reggio Emilia: Fabio Quartieri, Nicola Bottoni, Matteo Iori; C.D.C. Montevergine, Mercogliano: Francesco Solimene; Carlo Poma, Mantova: Patrizia Pepi, Albino Reggiani; Cattinara, Trieste: Massimo Zecchin; Civile Ferrari, Castrovillari: Giovanni Bisignani; Conegliano Hospital, Conegliano: Roberto Mantovan, Giuseppe Allocca, Nadir Sitta, Luigi Rivetti. Desio Hospital, Desio: Giuseppe Mantovani, Giulia Balestri; Destra Secchia, Pieve di Coriano: Daniela Pozzetti, Mario Pasqualini; Ferrari, Casarano: Donato Melissano, Giovanni Carlo Piccinini; Ferrarotto, Catania: Valeria Calvi, Francesco Platania; Fondazione di Ricerca e Cura Giovanni Paolo II, Campobasso: Matteo Santamaria, Parisi Quintino, Celestino Sardu, Loredana Messano; FTGM Pisa, Pisa: Marcello Piacenti, Luca Panchetti, Umberto Startari, Andrea Rossi; Gemelli, Roma: Maria Lucia Narducci, Gemma Pelargonio, Francesco Raffaele Spera; Gruppo Villa Maria Care&Reserarch, Cotignola: Saverio Iacopino, Pasquale Filannnino, Paolo Artale; INRCA, Ancona: Marinella Marini, Lorenzo Pimpini; Lodi Hospital, Lodi: Fabio Lissoni, Stefano Tinelli, Giulia Caetani, Egidio Marangoni, Sara Morbio; Macerata Hospital, Macerata: Gianluigi Morgagni; Manzoni, Lecco: Camillo Gerosa; Antonio Pani, Roberta Brambilla; Maria Vittoria, Torino: Massimo Giammaria, Claudia Amellone, Maria Teresa Lucciola; Mater Salutis, Legnago: Gabriele Zanotto, Emanuela Visentin, Davide Sandrini; Misericordia, Grosseto: Gennaro Miracapillo, Luigi Addonisio, Marco Breschi, Francesco De Sensi; Monaldi Ospedalieri, Napoli: Antonio D'Onofrio, Multimedica, Milano: Edoardo Gronda, Antonio Sagone, Ruggiero Donatella, Tiziana Staine; OO.RR. S.Giovanni di Dio e Ruggi D'Aragona, Salerno: Michele Manzo, Gaetana Melchiorre; S. Croce, Cuneo: Antonello Vado, Gianpaolo Baccari, Cecilia Goletto; Ospedale Apuane, Massa: Giuseppe Arena, Massimo Ratti, Vincenzo Borrello, Iacopo Bertolozzi; Ospedale dei Colli - Monaldi Sun, Napoli: Gerardo Nigro, Ernesto Ammedola, Vincenzo Russo, Anna Rago; Ospedale dell'Angelo, Mestre: Elena Marras, Sakis Themistoclakis; Ospedale di Circolo e Fond. Macchi Cardiologia 1, Varese: Fabrizio Caravati; Ospedale di Circolo e Fond. Macchi Cardiologia 2, Varese: Paolo Bonfanti; Ciriè Hospital, Ciriè: Gaetano Senatore, Giuseppe Trapani, Claudia Amellone, Marco Giuggia; Sacco Hospital, Milano: Giovanni Forleo, Leonida Lombardi; Versilia Hospital, Camaiore: Marco Tullio Baratto, Enrica Talini; P.O. "V. Cervello", Palermo: Paola Vaccaro, Vincenzo Lo Giudice; P.O. San Paolo, Bari: Pasquale Cardarola, Luigi Mancini, Cosimo Campanella, Manuela Resta; P.O.S. Timoteo, Termoli: Emilio Musacchio; Policlinico Federico II, Napoli: Antonio Rapacciuolo, Francesca Esposito, Gianluigi Iovino, Alessia Agresta; Pugliese – Ciaccio, Catanzaro: Giampiero Maglia; Ramazzini, Carpi: Elia De Maria, Ambra Borghi; Riuniti Lancisi, Ancona: Alessandro Capucci, Mario Luzi; S. Antonio Abate, Gallarate: Daniela Orsida; S. Gerardo, Monza: Giovanni Rovaris; S. Maria del Carmine, Rovereto: Massimiliano Maines; S.Maria Nuova, Firenze: Maria Giaccardi, Alessandro Paoletti Perini, Lanfranco Fratoni, Andrea Giomi; S.S. Costa e Damiano, Pescia: Stefano Di Marco, Stefano Gabbani; San Filippo Neri, Roma: Carlo Pignalberi, Loredana Morichelli, Antonio Porfili, Laura Quarta, Anna Sassi; San Giuseppe, Empoli: Attilio Del Rosso, Vincenzo Guarnaccia; San Raffaele, Milano: Paolo Della Bella, Pasquale Vergara, Caterina Bisceglia; Santa Chiara, Trento: Massimiliano Marini, Alessio Coser, Fabrizio Guarracini; Santa Maria della Misericordia, Urbino: Maurizio Mezzetti, Iacopo Ciccarelli, Andrea Giovagnoli; Sant'Anna, Como: Stefano Pedretti, Chiara Belvito, Carlo Piemontese; Sant'Orsola, Bologna: Mauro Biffi, Matteo Ziacchi, Igor Diemberger, Cristian Martignani; Spedali Civili, Brescia: Antonio Curnis, Luca Bontempi, Manuel Cerini; Vimercate Hospital, Vimercate: Riccardo Sacchi, Maximo Panigada, Andrea Colombo; Vito Fazzi, Lecce: Ennio C. Pisanò, Tiziana Mazzella.
